# The Transcription Factor OsWRKY45 Negatively Modulates the Resistance of Rice to the Brown Planthopper *Nilaparvata lugens*

**DOI:** 10.3390/ijms17060697

**Published:** 2016-05-31

**Authors:** Jiayi Huangfu, Jiancai Li, Ran Li, Meng Ye, Peng Kuai, Tongfang Zhang, Yonggen Lou

**Affiliations:** State Key Laboratory of Rice Biology, Institute of Insect Sciences, Zhejiang University, Hangzhou 310058, China; luckyhfjy@163.com (J.H.); jiancai.2007@163.com (J.L.); li-ran@hotmail.com (R.L.); hmily890404@126.com (M.Y.); kpchen7493@163.com (P.K.); aimarjia@gmail.com (T.Z.)

**Keywords:** rice, WRKY transcription factor, OsWRKY45, *Nilaparvata lugens*, herbivore-induced plant defense, H_2_O_2_, ethylene

## Abstract

WRKY transcription factors play a central role not only in plant growth and development but also in plant stress responses. However, the role of WRKY transcription factors in herbivore-induced plant defenses and their underlying mechanisms, especially in rice, remains largely unclear. Here, we cloned a rice WRKY gene *OsWRKY45*, whose expression was induced by mechanical wounding, by infestation of the brown planthopper (BPH, *Nilaparvata lugens*) and by treatment with jasmonic acid (JA) or salicylic acid (SA). The antisense expression of *OsWRKY45* (as-*wrky*) enhanced BPH-induced levels of H_2_O_2_ and ethylene, reduced feeding and oviposition preference as well as the survival rate of BPH, and delayed the development of BPH nymphs. Consistently, lower population densities of BPH on as-*wrky* lines, compared to those on wild-type (WT) plants, were observed in field experiments. On the other hand, as-*wrky* lines in the field had lower susceptibility to sheath blight (caused by *Rhizoctonia solani)* but higher susceptibility to rice blast (caused by *Magnaporthe oryzae*) than did WT plants. These findings suggest that OsWRKY45 plays important but contrasting roles in regulating the resistance of rice to pathogens and herbivores, and attention should be paid if OsWRKY45 is used to develop disease or herbivore-resistant rice.

## 1. Introduction

To protect themselves from attack by biotic stresses, such as pathogens and herbivores, plants have evolved constitutive and induced defenses [1,2,3]. Induced plant defense begins with the recognition of pathogen- or herbivore-associated signals, followed by the elicitation of a defense-related signaling network consisting mainly of jasmonic acid (JA)-, salicylic acid (SA)-, ethylene- and H_2_O_2_-mediated pathways and the up-regulation of transcript levels of defense-related genes and the production of defense compounds [4]. During this induction process, transcription factors (TF) perform a vital role [5,6,7,8].

WRKY TFs, which are named after the highly conserved domain WRKYGQK at the N-terminus and which specifically bind the *cis*-elements (TTGACC/T; W-box) in the promoter regions of their target genes, make up one of the largest families of TFs in plants [9,10,11]. At the C-terminus, WRKY TFs always contain a zinc-finger-like motif: Cx_4-5_Cx_22-23_HxH or Cx_7_Cx_23_HxC. Based on the features of zinc-finger motifs and the number of WRKY domains, both of which are very important for the interaction of WRKT TFs with their target genes, WRKY TFs are classed into three groups [9]. WRKYs have also been reported to play important roles in plant defense responses to pathogens and herbivores besides their central role in growth, development and abiotic stress responses of plants [9,12,13]. In *Arabidopsis*, for example, *AtWRKY33*, *AtWRKY38*, *AtWRKY62* and *AtWRKY70* are involved in plant defense against different pathogens [14,15,16]. In rice (*Oryza sativa*), *OsWRKY13*, *OsWRKY45*, *OsWRKY53* and *OsWRKY89* regulate plant pathogen resistance, and *OsWRKY53*, *OsWRKY70* and *OsWRKY89* also mediate herbivore resistance [6,7,17,18,19,20,21]. Studies on mechanisms underlying the WRKY-mediated plant resistance revealed that WRKYs are involved in the defense-related signaling network by acting up- and down-stream of receptors, protein kinases and signal molecules [7,11,22]. In *Nicotiana attenuata*, for instance, *NaWRKY3* and *NaWRKY6* control the production of herbivore-elicited JA and JA-Ile/-Leu, which in turn shapes herbivore-induced defenses [23]. Recently, it has been reported that OsWRKY70 prioritizes rice defense over growth by positively modulating JA and negatively mediating gibberellin (GA) biosynthesis upon infestation by the rice stem borer *Chilo suppressalis* [6]. Moreover, OsWRKY53 is a negative modulator of OsMPK3/OsMPK6 and thereby functions as an early suppressor of defense responses in rice [7]. While the roles of WRKYs in plant defense responses against biotic stresses are known, the underlying molecular mechanisms and the roles of different WRKYs in this process remain largely unclear.

Rice, one of the most important food crops worldwide, suffers severely from insect pests [24]; the brown planthopper (BPH), *Nilaparvata lugens* (Stål), is one of the most important. BPH feeds on phloem sap and causes a reduction in many physiological and biochemical parameters, such as plant growth and photosynthetic rate, which subsequently results in yield loss [25]. BPH attack on rice elicits the production of multiple signals, such as JA, SA and H_2_O_2_, which in turn modulate defense responses, such as the release of herbivore-induced volatiles, and the increase in the activity of trypsin protease inhibitors [5,26,27,28,29]. Moreover, it has been found that JA- and ethylene-mediated signaling pathways both negatively and, in the case of the H_2_O_2_ pathway, positively regulate the resistance of rice to BPH [5,27,30]. Given the importance of WRKYs in plant defense responses, we cloned the rice WRKY TF *OsWRKY45*, which belongs to group III of WRKY TFs and contains one conserved WRKY domain and a Cx_7_Cx_23_HxC-type zinc-finger-like motif, and clarified its role in defense responses of rice infested by BPH. OsWRKY45 localizes to the nucleus and plays a pivotal role in BTH-elicited disease resistance by functioning downstream of SA signaling [17,18,31,32]. The over-expression of *OsWRKY45* enhances the resistance of rice to multiple biotrophic and hemibiotrophic pathogens, such as *Magnaporthe oryzae* and *Xanthomonas oryzae pv. Oryzae* (*Xoo*), but not to a nectrophic pathogen *Rhizoctonia solani* [17,32]. Moreover, OsWRKY45 is induced by herbivore infestation [7]. However, whether and how OsWRKY45 mediates herbivore-induced defense responses in rice is unknown.

Here, we show that *OsWRKY45* is induced by BPH infestation, mechanical wounding and JA or SA treatment. By combining data from molecular biology, reverse genetics, chemical analysis and bioassays, we show that silencing OsWRKY45 enhances both the levels of BPH-induced H_2_O_2_ and ethylene, and the extent of resistance in rice to BPH. These findings suggest that *OsWRKY45* is a negative modulator of herbivore-elicited defense responses in rice.

## 2. Results

### 2.1. Isolation and Characterization of OsWRKY45

Our previous research found that infestation of the rice striped stem borer *Chilo suppressalis* induced the expression of *OsWRKY45* [7]. The full-length cDNA of *OsWRKY45* from a *japonica* type variety Xiushui 11 was obtained via reverse transcription PCR. The sequence of *OsWRKY45* cloned here, including an open reading frame of 981 bp (Supplementary Figure S1), is 100% and 98.16%, respectively, identical to *OsWRKY45-1* (*japonica*-derived *WRKY45*) and *OsWRKY45-2* (*indica*-derived *WRKY45*) (Supplementary Figure S2), both of which is a pair of alleles and have been found to play different roles in biotic defense responses [18]. Phylogenetic analysis revealed that OsWRKY45 is homologous to ZmWRKY45 in *Zea mays*, to BdWRKY20 in *Brachypodium distachyon* [33], to OsWRKY79 in rice [34], and to AtWRKY54 and AtWRKY70 in *Arabidopsis thaliana* [35] (Supplementary Figure S3); all of these proteins share 47%, 43%, 34%, 35% and 39% amino acid sequence identity with OsWRKY45, respectively.

**Figure 1:**
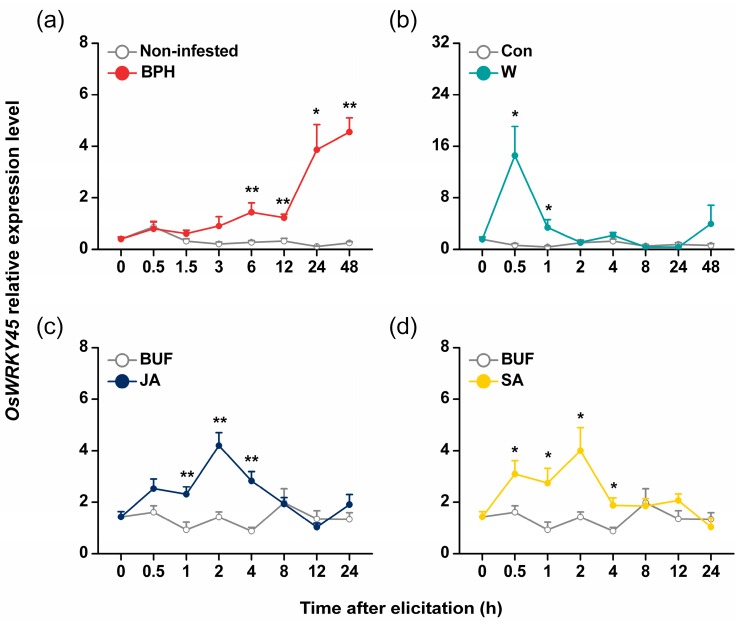
Relative expression level of *OsWRKY45* in rice after various treatments. Mean transcript levels (±SE, *n* = 5) of *OsWRKY45* in stems of rice plants that were infested by rice brown planthopper (BPH) (**a**), mechanically wounded (W) (**b**) and treated with jasmonic acid (JA) (**c**) or salicylic acid (SA) (**d**). BUF, buffer; Con, non-manipulated plants; non-infested, plant stems were individually confined with an empty glass cage. Transcript levels were analyzed by quantitative RT-PCR. Asterisks indicate significant differences in transcript levels between treatments and controls (* *p* < 0.05, ** *p* < 0.01, Student’s *t*-tests).

Quantitative real-time PCR analysis of *OsWRKY45* revealed its low constitutive expression; however, BPH infestation resulted in the significant induction of *OsWRKY45* expression-levels reached a maximum at 48 h (Figure 1a). Mechanical wounding also significantly induced the expression of *OsWRKY45*, peaking at 0.5 h (Figure 1b). JA and SA treatment elicited the expression of the gene constantly and significantly (Figure 1c,d). These data suggest that *OsWRKY45* might be involved in rice defense resistance to BPH.

### 2.2. Antisense Expression of OsWRKY45

**Figure 2:**
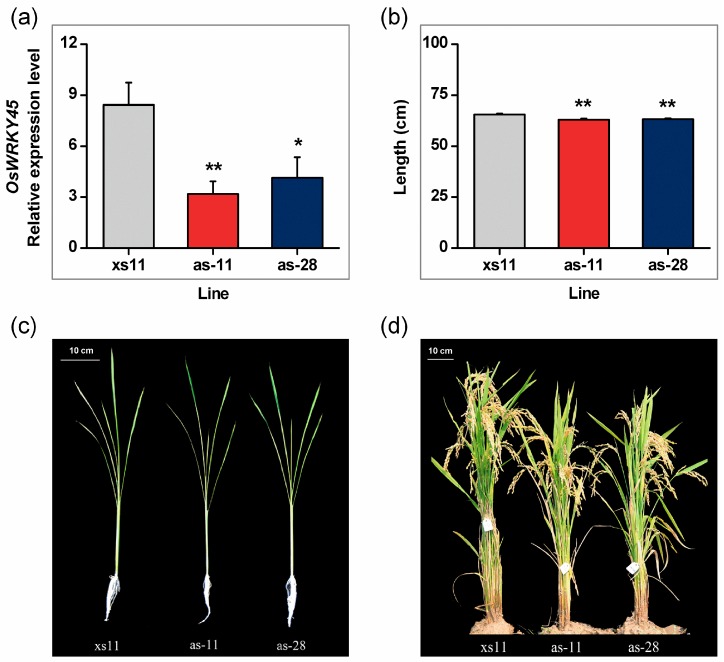
*OsWRKY45* expression levels and growth phenotypes of as-*wrky* and wild-type (WT) plants. (**a**) Mean levels (±SE, *n* = 5) of *OsWRKY45* transcripts in as-*wrky* lines and WT plants that were wounded for 0.5 h. Transcript levels were analyzed by qRT-PCR; (**b**) mean length (±SE, *n* = 20) of 40-day-old as-*wrky* and WT plants; (**c**) 30-day-old seedlings of as-*wrky* and WT lines in the greenhouse; (**d**) plants of as-*wrky* and WT lines at the heading stage in the field. Asterisks indicate significant differences in as-*wrky* lines compared to WT plants (* *p* < 0.05, ** *p* < 0.01, Student’s *t*-tests).

To investigate the role of OsWRKY45 in herbivore-induced rice defense, three T2 homozygous OsWRKY45-antisensed lines (as-*wrky* lines: as-11, as-24, as-28) were obtained; two of the three lines (as-11 and as-28) contain a single T-DNA insertion (Supplementary Figure S4). Transcription analysis demonstrated that mRNA levels of *OsWRKY45* in the two as-*wrky* lines, as-11 and as-28, were 38% and 49% of those in wild-type plants at 0.5 h after wounding (Figure 2a). In rice, nucleotide sequences of three genes, *OsWRKY54* (62%, accession no. Os05g40080), *OsWRKY48* (61%, accession no. Os05g40060) and *OsWRKY15* (58%, accession no. Os01g46800), have the highest similarity to that of *OsWRKY45*. By comparing sequences of these three genes with that used for antisense transformation, no more than 19 consecutive identical nucleotides were found (Supplementary Figure S5). This suggests that the antisense sequence is highly specific, and that the expression of any other rice genes should not be co-silenced by the antisense construct. When planted in a greenhouse or the paddy, as-*wrky* lines showed slight growth retardation, especially at the ripe stage in the field (Figure 2c,d). At 40-day-old, the total length of as*-wrky* plants decreased by approximately 4% compared to the length of WT plants (Figure 2b).

### 2.3. Silencing OsWRKY45 Enhances Levels of BPH-Induced Ethylene and H_2_O_2_ but Not of JA and SA

**Figure 3:**
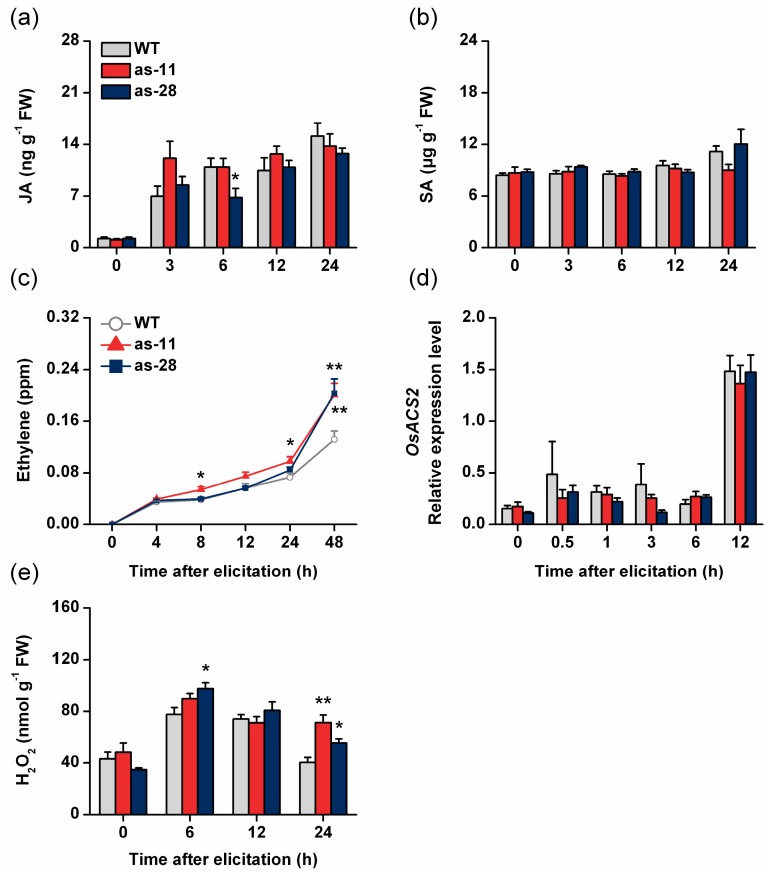
*OsWRKY45* mediatesthe levels of BPH-induced ethylene and H_2_O_2_ but not JA and SA. Mean concentrations (±SE, *n* = 5) of jasmonic acid (JA) (**a**), salicylic acid (SA) (**b**) and ethylene (**c**) in as-*wrky* and wild-type (WT) plants that were individually infested with 15 gravid brown planthopper (BPH) females; (**d**) Mean transcriptlevels (±SE, *n* = 5) of *OsACS2* in as-*wrky* and WT plants that were individually infested with 15 gravid BPH females; (**e**) Mean concentrations (±SE, *n* = 5) of H_2_O_2_ in as-*wrky* and wild-type WT plants that were individually infested with 15 gravid BPH females. Asterisks indicate significant differences in as-*wrky* lines compared with WT plants (* *p* < 0.05, ** *p* < 0.01, Student’s *t*-tests).

Signal molecules, such as JA, SA, ethylene and H_2_O_2_, have been reported to play major roles in herbivore-induced defense responses in many plant species, including rice [36,37,38]. Moreover, JA and ethylene signaling pathways negatively regulate the resistance of rice to BPH, whereas H_2_O_2_ seem to positively mediate resistance to BPH [7,27,30]. To determine whether *OsWRKY45* influences the biosynthesis of BPH-elicited JA, SA, ethylene and H_2_O_2_ and thus regulates the resistance of rice to BPH, levels of these signals were investigated in WT plants and as-*wrky* lines. Infestation by female BPH adults elicited the production of JA and SA (weakly at the later time points) in both WT and as-*wrky* plants (Figure 3a,b). However, there was no significant difference in basal or BPH-induced levels of JA and SA between WT and as-*wrky* plants, suggesting that OsWRKY45 does not affect the production of BPH-induced JA and SA (Figure 3a,b).

Compared with those in WT plants, levels of BPH-induced ethylene were significantly higher in the as-*wrky* lines (Figure 3c). We also measured the transcript level of the *OsACS2* gene, which encodes an1-aminocyclopropane-1-carboxylic acid synthase (ACS), a speed-limiting enzyme for ethylene biosynthesis [30], and found that the constitutive and induced mRNA levels of this gene did not differ between the WT plants and as-*wrky* lines (Figure 3d). No difference was observed in basal H_2_O_2_ levels between the as-*wrky* lines and WT plants, however, after BPH infestation, H_2_O_2_ levels were obviously enhanced (by about 15%–26% and 37%–77% at 6 and 24 h, respectively) in as-*wrky* lines compared with WT plants (Figure 3e). The data suggest that silencing OsWRKY45 significantly increased the levels of BPH-induced ethylene and H_2_O_2_ but did not influence the biosynthesis of JA and SA.

### 2.4. OsWRKY45 Negatively Mediates Rice Resistance to BPH

**Figure 4:**
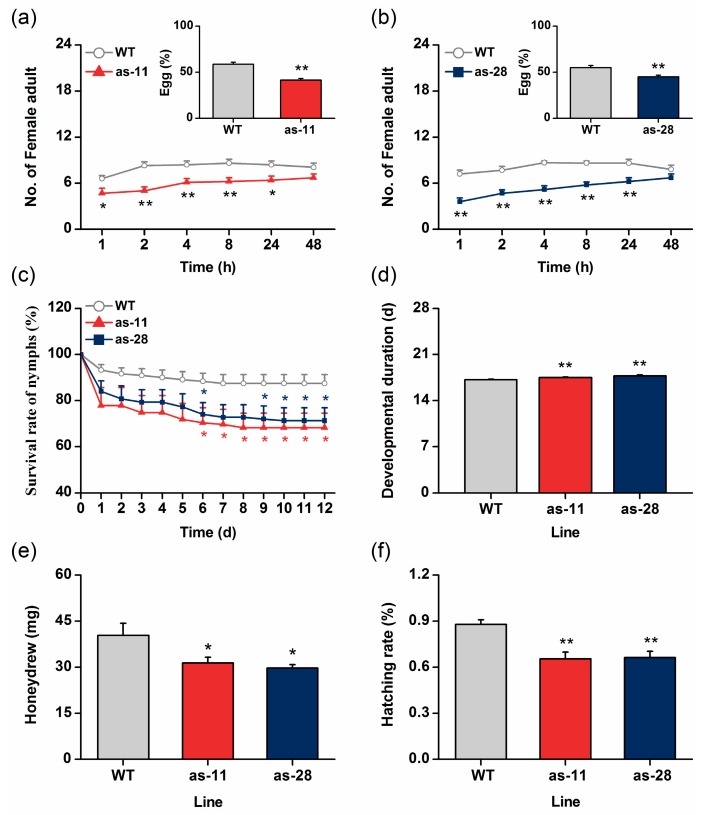
*OsWRKY45* negatively mediates the resistance ofrice to the brown planthopper (BPH). (**a**,**b**) Mean number of BPH adult females per plant (±SE, *n* = 10) on pairs of plants, an as-11 plant *versus* a wild-type (WT) plant and an as-28 plant *versus* a WT plant. Insets: Mean percentage (±SE, *n* = 10) of BPH eggs per plant on pairs of plants as mentioned above; (**c**) mean survival rate (±SE, *n* = 10) of BPH nymphs that fed on as-*wrky* lines and WT plants for 1–12 days after the release of insects; (**d**) mean developmental duration (±SE, *n* = 90–130) of immature-stage BPH nymphs feeding on as-*wrky* lines and WT plants; (**e**) mean mass (±SE, *n* = 25) of honeydews excreted by a female BPH adult per day on as-*wrky* lines and WT plants; (**f**) mean hatching rate (±SE, *n* = 10) of BPH eggs on as-*wrky* lines and WT plants. Asterisks indicate significant differences in as-*wrky* compared with WT plants (* *p* < 0.05, ** *p* < 0.01, Student’s *t*-tests).

We investigated whether silencing *OsWRKY45* has an effect on BPH preference and performance. BPH adult females preferred to feed and oviposit on WT plants rather than on as-*wrky* lines (Figure 4a,b). The number of BPH eggs on transgenic lines, as-11 and as-28, was decreased by 17% and 10%, respectively, compared to those on WT plants. In accordance with the feeding preference mentioned as above, BPH female adults fed on as-*wrky* lines excreted significantly less honeydew, an indicator of food intake, than those fed on WT plants (Figure 4e). Silencing of OsWRKY45 also reduced the survival rate of BPH nymphs (by 22% and 18% on as-11 and as-28 plants at 12 days, respectively; Figure 4c) and the hatching rate of BPH eggs (by 22% and 21%, respectively; Figure 4f). In addition, silencing OsWRKY45 significantly prolonged the developmental duration of the immature stage in BPH (Figure 4d). These data demonstrate that OsWRKY45 negatively mediates the resistance in rice to BPH.

### 2.5. Effect of OsWRKY45 on Multitrophic Interactions in the Field

**Figure 5:**
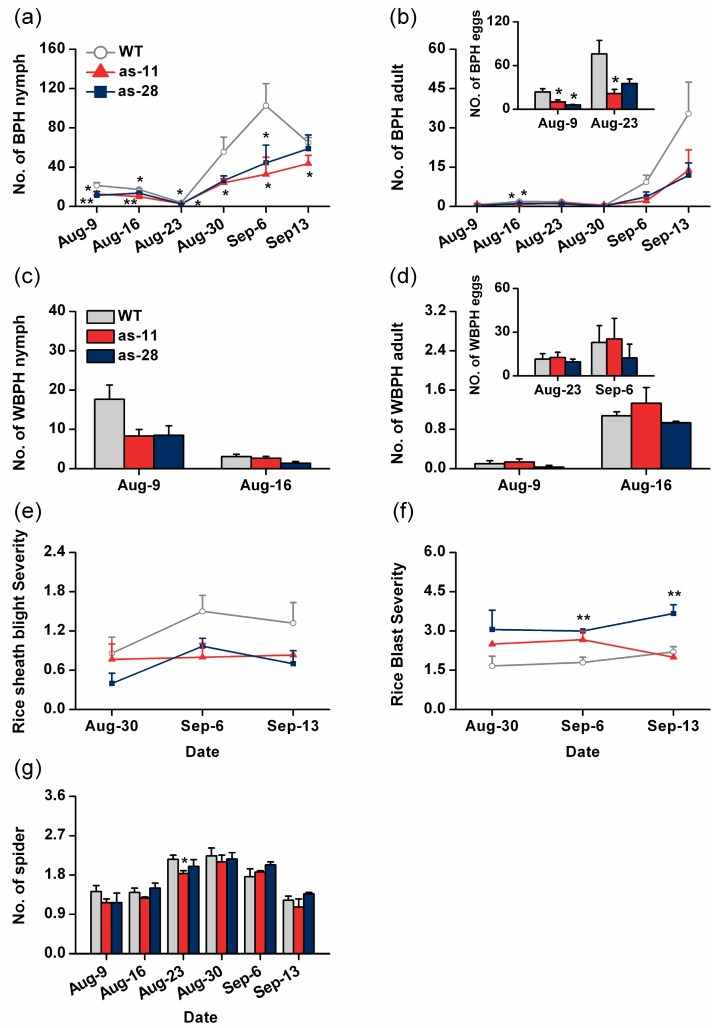
Planthopper abundance, spider numbers and disease severity on as*-wrky* and wild-type (WT) plants in a field experiment. Mean numbers (±SE, *n* = 3) of BPH nymphs (**a**) and adults (**b**) per two hills of plants on as*-wrky* and WT lines; Inset: mean numbers (±SE, *n* = 20) of BPH eggs per plant on as*-wrky* and WT lines; mean numbers (±SE, *n* = 3) of WBPH nymphs (**c**) and adults (**d**) per two hills of plants on as*-wrky* and WT lines; Inset: mean numbers (±SE, *n* = 20) of BPH eggs per plant on as*-wrky* and WT lines. Mean severity (±SE, *n* = 3) of rice sheath blight (**e**) and rice blast (**f**) on as*-wrky* and WT plants; (**g**) mean numbers (±SE, *n* = 3) of spiders per two hills on plants on as*-wrky* and WT lines. Asterisks indicate significant differences in as*-wrky* compared with WT plants (*, *p*< 0.05, ** *p*< 0.01, Student’s *t*-tests).

To determine whether OsWRKY45 could influence the performance of rice pests and their natural enemies in nature, we performed a survey of the population dynamics of these pests and of their natural enemies on WT plants and on as-*wrky* lines in the field. During the field experiment, BPH, the major herbivore in this year, and another piercing-sucking insect pest, the white-backed planthopper (WBPH), *Sogatella furcifella*, are insect pests that we regularly observed. Natural enemies were mainly predatory spiders, of which *Pirata subpiraticus*, *Misumenops tricuspidatus*, *Pardosa pseudoannulata* and *Tetragnatha maxillosa* were dominant species. Rice diseases were mainly blast, caused by *M. oryzae*, and sheath blight, caused by *R. solani*. Consistent with the results in the lab, the number of BPH adults, nymphs and eggs, especially nymphs and eggs, on as-*wrky* lines in the field were significantly lower than the number on WT plants (Figure 5a,b). Unlike the numbers of BPH nymphs, adults and eggs, the numbers of nymphs, adults and eggs of WBPH show no significant difference between as-*wrky* lines and WT plants, although on as-*wrky* lines, the numbers were decreasing (Figure 5c,d). The number of spiders on each plant type was also the same except on August 23 when the number of spiders on one as*-wrky* line as-11 was obviously lower than that on WT plants (Figure 5g). Consistent with Simono *et al.* [17], compared to WT plants, as-*wrky* lines showed less resistance to rice blast but more resistance to rice sheath blight (Figure 5e,f).

## 3. Discussion

In this study, we investigated the role of OsWRKY45 in herbivore-induced defense responses in rice. By combining data from molecular biology, reverse genetics, chemistry and bioassays, we found that BPH infestation induced the expression of *OsWRKY45* (Figure 1a). Moreover, silencing *OsWRKY45* enhanced the levels of BPH-elicited ethylene and H_2_O_2_ but did not influence the production of JA and SA (Figure 3); in the end, the resistance of rice to BPH increased (Figure 4). These data demonstrate that *OsWRKY45* negatively modulates the resistance of rice to BPH.

The OsWRKY45-dependent branch is an important pathway off the main SA pathway [32]. OsWRKY45 could be induced by pathogen infection as well as by treatment with SA and BTH but not with MeJA [32]. Here we found that BPH infestation and JA treatment could also elicit the expression of OsWRKY45 (Figure 1). This suggests that OsWRKY45 is involved in multiple biotic stress responses. The fact that JA induced the expression of OsWRKY45 but not of MeJA indicates that in rice, exogenous MeJA functions differently from exogenous JA. Different biological functions of JA and MeJA in plants have been reported in many studies [39].

WRKYs can regulate the production of JA, JA-Ile, SA, ethylene and H_2_O_2_ by directly or indirectly influencing the activity of related enzymes [15,23,40,41,42]. In *Arabidopsis thaliana*, for example, by binding to W-boxes in the promoters of *ACS2* and *ACS6*, WRKY33 mediates transcript levels of the two genes [42,43]. OsWRKY53 was reported to negatively modulate the biosynthesis of herbivore-induced JA, JA-Ile and ethylene as well as the mRNA levels of JA and ethylene biosynthesis-related genes, whereas it had a positive effect on the level of SA after SSB infestation [7]. In *Tamarix hispida*, the WRKY gene *ThWRKY4* enhances the activity of superoxide dismutase and peroxidase, thus decreasing levels of O_2_^−^ and H_2_O_2_ [44]. For OsWRKY45, it has been reported that the over-expression of *OsWRKY45* increased the levels of H_2_O_2_ in plants when they were infected with *M. oryzae* [17]. Moreover, OsWRKY45 positively modulated the biosynthesis of JA when plants were infected by a parasitic plant species, *Striga hermonthica* [45]; however, it negatively mediated JA accumulation in *Xoo*-infected plants [18]. We found that silencing OsWRKY45 enhances levels of BPH-elicited H_2_O_2_ and ethylene but did not influence basal and induced levels of JA and SA (Figure 3). These data demonstrate that OsWRKY45 can regulate JA, ethylene and H_2_O_2_ pathways in rice and that its effect on these pathways is dependent on the specific interaction of rice with the biotic stress. Interestingly, silencing OsWRKY45 did not enhance the elicited expression of *OsACS2*, a gene encoding a key enzyme in ethylene biosynthesis, although it did enhance the BPH-induced ethylene level (Figure 3d). This finding suggests that OsWRKY45 might mediate ethylene biosynthesis through other key regulators. Further research should elucidate how OsWRKY45 modulates the biosynthesis of these signal molecules.

JA and ethylene signaling pathways have been found to negatively mediate the resistance of rice to BPH [27,30], whereas the H_2_O_2_ pathway positively mediates resistance to BPH [7]. Thus, the fact that the antisense expression of *OsWRKY45* enhanced the resistance of rice to BPH in the laboratory and field is probably due to high H_2_O_2_ levels (Figure 3e). Consistent with the results reported in Shimono *et al.* [17], our field experiment also found that as*-wrky* lines, compared to WT plants, had more damage from rice blast and less damage from rice sheath blight (Figure 5e,f). Interestingly, we observed that as*-wrky* plants showed some growth retardation compared with WT plants, especially in the field (Figure 2). This disparity differed from what Simono *et al.* (2007) found in the greenhouse and growth chamber, namely, a dwarfed growth phenotype of rice lines that over-expressed *OsWRKY45* [32]. This inconsistency is probably related to the environmental conditions (biotic and abiotic factors) under which the plants were growing, as Simono *et al.* (2007) argued [32].

We found that BPH infestation induced the expression of *OsWRKY45* and silencing *OsWRKY45* enhanced the resistance of rice to BPH; this result demonstrates that BPH could overcome rice resistance by secreting effectors that activate OsWRKY45. Increasing evidence shows that defense responses of plants induced by piercing-sucking insects, including BPH, are similar to those elicited by pathogens [46]; moreover, OsWRKY45 is responsive to pathogen infection and plays a vital role in regulating the resistance of rice to pathogens [17,32]. Thus, an alternative explanation for the BPH induction of *OsWRKY45* is that plants might misperceive BPH infestation as pathogen infection and then activate defense responses to pathogens that do not respond effectively to BPH. How BPH infestation induces the expression of *OsWRKY45* should be explored in the future.

Rice plants over-expressing *OsWRKY45* have been observed to resist multiple pathogens and to show only small fitness costs [17,32,47]. Therefore, developing disease-resistant rice varieties by optimizing the expression of *OsWRKY45* (minimizing the fitness cost) or by pathogen-responsive expression of *OsWRKY45* has been put forward [47,48,49]. However, based on the results reported here, attention should be paid to these disease-resistant varieties as they may incur severe damage by BPH, one of the most important rice pests in Asia.

## 4. Materials and Methods

### 4.1. Plant Growth

The rice genotypes used in this study were *japonica* type variety Xiushui 11 (wild-type, WT) and as-*wrky* transgenic lines (see below). Pre-germinated seeds of the different lines were cultured in plastic bottles (diameter 8 cm, height 10 cm) in a greenhouse (28 ± 2 °C, 14 h light, 10 h dark). Ten days later, the seedlings were transferred to 20-L hydroponic boxes with a rice nutrient solution [50]. After 30 days, seedlings were transferred to individual 500 mL hydroponic opaque plastic pots (diameter 8 cm, height 10 cm), each with one or two plants (one WT plant and one as-*wrky* transgenic line plant). Plants were used for experiments 4–5 days after transplanting.

### 4.2. Insects

Original colonies of BPH were obtained from rice fields in Hangzhou, China, and maintained on a BPH-susceptible rice variety TN1 in a climate chamber that was maintained at 26 ± 2 °C, with a 12 h light phase and 80% relative humidity.

### 4.3. Isolation and Characterization of OsWRKY45 cDNA

The full-length cDNA of *OsWRKY45* was PCR-amplified. The primers WRKY45-F (5′-TCGGTGGTCGTCAAGAACC-3′) and WRKY45-R (5′-AAGTAGGCCTTTGGGTGCTT-3′) were designed based on the sequence of rice *OsWRKY45* (TIGR ID Os05g25770). The PCR products were cloned into the Pmd19-T vector (TaKaRa, Dalian, China) and sequenced.

### 4.4. Phylogenetic Analysis

For the phylogenetic analysis, the characterized WRKYs from different species were selected and the program MEGA 6.0 [51] was used. The protein sequences aligned using the ClustalW method in MEGA 6.0 (pairwise alignment: gap opening penalty 10, gap extension penalty 0.1; multiple alignments: gap opening penalty 10, gap extension penalty 0.2, protein weight matrix using Gonnet). The residue-specific and hydrophilic penalties were on, and the end gap separation and the use negative separation matrix were off. The gap separation distance was 4, and the delay divergence cutoff (percentage) was 30. This alignment was then used to generate an unrooted tree with statistical tests (parameters for phylogeny reconstruction were neighbor-joining method [52] and bootstrap, *n* = 1000, amino acid, Poisson model, rates among sites: uniform rates gaps/missing, data treatment: complete deletion, traditional tree without modification for graphics) using MEGA 6.0.

### 4.5. qRT-PCR

For qRT-PCR analysis, five independent biological replicationswere used. Total RNA was isolated using the SV Total RNA Isolation System (Promega, Madison, WI, USA) following the manufacturer’s instructions. One μg of each total RNA sample was reverse transcribed using the PrimeScript RT-PCR Kit (TaKaRa, Dalian, China). The qRT-PCR assay was conducted on CFX96 Real-Time system (Bio-RAD, Hercules, CA, USA) using the SsoFast™ probes supermix (Bio-RAD, Hercules, CA, USA, http://www.bio-rad.com/). A linear standard curve, threshold cycle number *versus* log (designated transcript level), was built using a series concentrations of a specific cDNA standard. Relative levels of the transcript of the target gene in tested samples were calculated according to the standard curve. To normalize cDNA concentrations in samples, a rice housekeep gene actin *OsACT* (accession No. Os03g50885) was used as an internal standard. The primers and probes of all tested genes used for qRT-PCR were provided in Supplemental Table S1.

### 4.6. Generation and Characterization of Transgenic Plants

A 466 bp portion of *OsWRKY45* Cdna (Supplemental Figure S5) was cloned into the pCAMBIA-1301 transformation vector (Supplemental Figure S6), yielding an antisense construct. This vector was used to transform rice variety XS11 using an *Agrobacterium*-mediated transformation system. Rice transformation, screening of the homozygous T_2_ plants and identification of the number of insertions followed the same method described before [27]. Two T2 homozygous lines, as-11 and as-28, each with a single insertion, were chosen and used in subsequent experiments.

### 4.7. Plant Treatments

For mechanical wounding, plants (one per pot) were individually damaged on the lower part of the stems (about 2 cm long) using a needle, each with 200 pricks. Non-manipulated plants were used as controls. For BPH treatment, plants (one per pot) were individually infested with 15 gravid BPH females that were confined in a glass cage (diameter 4 cm, height 8 cm, with 48 small holes, diameter 0.8 mm). Control plants were covered with empty glass cages (non-infested). For JA and SA treatment, each plant was sprayed with 2 mL of JA (100 µg·mL^−1^) or SA (70 µg·mL^−1^) in 50 mM sodium phosphate buffer (pH 8, with 0.01% Tween). Plants sprayed with 2 mL of the buffer were used as controls.

### 4.8. JA and SA Analysis

Plants (one per pot) of the two transgenic lines, as-11 and as-28, and the WT line were randomly assigned to BPH and non-infested treatment. The stems were harvested at 0, 3, 6, 12, 24 and 48 h after BPH infestation. Samples were ground in liquid nitrogen, and JA and SA were extracted with ethyl acetate spiked with labeled internal standards (D6-JA and D6-SA) and analyzed with a high-performance liquid chromatography/mass spectrometry/mass spectrometry system according to the method described in Lu *et al.* [30]. Five biological replications were used for each treatment at each time interval.

### 4.9. Hydrogen Peroxide Analysis

Plants (one per pot) of the two transgenic lines, as-11 and as-28, and the WT line were randomly assigned to BPH and non-infested treatment. The stems were harvested at 0, 3, 6, 12, 24 and 48 h after BPH infestation. The H_2_O_2_ concentrations were investigated using an Amplex^®^ Red Hydrogen Peroxide/Peroxidase Assay Kit (Invitrogen, Eugene, OR, USA), following the manufacturer’s instructions. Five biological replications were used for each treatment at each time interval.

### 4.10. Ethylene Analysis

Plants (one per pot) of the two transgenic lines, as-11 and as-28, and the WT line were randomly assigned to BPH and non-infested treatment; BPH-treated and control plants were individually covered with a sealed glass cylinder (diameter 4 cm, height 50 cm). Ethylene production was measured at 4, 8, 12, 24 and 48 h after the start of BPH feeding, using gas chromatography-mass spectrometry with standard gas ethylene [53]. Five biological replications were used for each treatment at each time interval.

### 4.11. BPH Bioassays in the Laboratory

To investigate the influence of antisense expression of OsWRKY45 on feeding and oviposition preference of BPH, two plants (a transgenic plant, as-11 or as-28, *versus* a WT plant) were confined within glass cylinders (diameter 4 cm, height 8 cm, with 48 small holes, diameter 0.8 mm). In each cylinder, 15 gravid BPH females were released. The number of BPH females on each plant was recorded at 1, 2, 4, 8, 24 and 48 h after the release of insects. Seventy-two hours after infestation, plants were dissected under a microscope and the number of BPH eggs on each plant was recorded. The experiment was replicated 10 times.

To determine whether as-*wrky* lines have an effect on BPH feeding, newly emerging adult BPH females were individually introduced into parafilm bags (5 cm × 5 cm), which were then fixed on the stem base of transgenic lines (as-11, as-28) and WT plants. Twenty-four h later, the amount of honeydew excreted by one female adult was weighed. The experiment was repeated 25 times.

To assess the survival rates and developmental durations of BPH nymphs on transgenic lines and WT plants, the basal stem of each plant (transgenic and WT plants) was covered in a glass cylinder into which 15 newly hatched BPH nymphs were released. The numbers of surviving BPH nymphs each day on each plant were recorded until they emerged as adults. The survival rates and developmental duration of immature stage of BPH nymphs were calculated. Ten replications were performed for this experiment.

To detect the impact of transgenic lines on the hatching rate of BPH eggs, 15 gravid BPH females were allowed to lay eggs on transgenic lines (as-11, as-28) and WT plants for 12 h, and then all the pests were removed. The numbers of freshly hatched BPH nymphs on plants were recorded every day until no new nymphs occurred for 3 consecutive days. Unhatched eggs were counted to determine the hatching rate on each plant. The experiment was repeated 10 times.

### 4.12. Field Experiment

To investigate whether the as-*wrky* lines influence the pest community in the field, we implemented a field experiment in Changxing, Zhejiang, China, from summer to autumn 2014. The testing plot was divided into nine blocks (6 m × 4.5 m), and each block was separated by a 0.5-m rice buffer zone. In the plot, three lines, the two as-*wrky* lines and their corresponding WT line (XS11), were randomly assigned to 9 blocks, each line with 3 independent replicate blocks. The number of nymphs, eggs, female and male adults of BPH and WBPH as well as the number of predatory spiders in each block were recorded once a week from 9 August to 13 September. Moreover, the severity of main diseases, including rice blast caused by *M. oryzae* and rice sheath blight caused by *R. solani,* was investigated at the peak of disease following the methods described in [54]. To determine the number of BPH eggs, we randomly sampled 20 plants per plot at each time interval and counted the total eggs on each plant using the same method as described in Xiang *et al.* [55]. To collect other data, we investigated 10 hills of plants in each plot at each time interval and recorded the number of insect pests and spiders as well as the severity of disease.

### 4.13. Data Analysis

Differences in mRNA levels of genes, plant heights, concentrations of JA, SA, ethylene and H_2_O_2_, the amount of honeydew, herbivore performance, and the number of insect pests and predatory spiders as well as the severity of disease, in different lines, treatments, or treatment times were determined by analysis of variance (or Student’s *t*-test for comparing two treatments). All tests were carried out with Statistica (SAS Institute, Inc., Cary, NC, USA, http://www.sas.com/).

## Supplementary Materials

Supplementary materials can be found at http://www.mdpi.com/1422-0067/17/6/697/s1.
