# Supplementary material for: The Transcription Factor OsWRKY45 Negatively Modulates the Resistance of Rice to the Brown Planthopper Nilaparvata lugens
Source: Int J Mol Sci. 2016 May 31;17(6):697. doi: 10.3390/ijms17060697 (PMC4926322; doi:10.3390/ijms17060697)
Supplement: Supplementary file 1 [file ijms-17-00697-s001.pdf]

Supplementary Materials: The Transcription Factor OsWRKY45 Negatively Modulates the Resistance of Rice to the Brown Planthopper *Nilaparvata lugens*

Jiayi Huangfu, Jiancai Li, Ran Li, Meng Ye, Peng Kuai, Tongfang Zhang and Yonggen Lou

```
1    ATGACGTCATCGATGTCGCCGGCGCCGGCGCCGGCGTACGCGCAGGTGATGGAGGACATG
1    M  T  S  S  M  S  P  A  P  A  P  A  Y  A  Q  V  M  E  D  M
61   GAGAAGGGGAAGGAGCTGGCGGCGCAGCTGCAGGGGCTCCTCCGCGACTCGCCGGAGGCC
21   E  K  G  K  E  L  A  A  Q  L  Q  G  L  L  R  D  S  P  E  A
121  GGCCGCTTCGTCGACCAGATTCTCCACACCTTCTCCCGGGCGATGCGGGCGCTCGACAAG
41   G  R  F  V  D  Q  I  L  H  T  F  S  R  A  M  R  A  L  D  K
181  GCGGCGGTCTCCGCCGCCGGAGGAGAAGGGTCGGAGGTGCAGAGCGAGGTCACCTGCGGG
61   A  A  V  S  A  A  G  G  E  G  S  E  V  Q  S  E  V  T  C  G
241  GGCGGGGCCAGCGCCGGCGGGAAGAGGAAAGCCCCCGCCGCCGACCGGAAGGCCAACTGC
81   G  G  A  S  A  G  G  K  R  K  A  P  A  A  D  R  K  A  N  C
301  CGCAGGAGGACGCAGCAATCGTCCGGGAATTCGGTGGTCGTCAAGAACCTCGACGACGGC
101  R  R  R  T  Q  Q  S  S  G  N  S  V  V  V  K  N  L  D  D  G
361  CAGGCATGGCGCAAGTACGGGCAGAAGGAGATCCAAAACTCCAAGCACCCAAAGGCCTAC
121  Q  A  W  R  K  Y  G  Q  K  E  I  Q  N  S  K  H  P  K  A  Y
421  TTCCGGTGCACGCACAAGTACGACCAGCTGTGCACGGCGCAGCGGCAGGTGCAGCGCTGC
141  F  R  C  T  H  K  Y  D  Q  L  C  T  A  Q  R  Q  V  Q  R  C
481  GACGACGACCCGGCGAGCTACAGGGTCACCTACATCGGCGAGCACACCTGCCGGGACCCG
161  D  D  D  P  A  S  Y  R  V  T  Y  I  G  E  H  T  C  R  D  P
541  GCCACCGCCCCCATCATCGCGGCGCACGTCATCCACCAGGTCGCCGCCGGCGACAACGAC
181  A  T  A  P  I  I  A  A  H  V  I  H  Q  V  A  A  G  D  N  D
601  GACGGCTGCGGCGGCCTCCAAGCGGGGTCCCGCCTCATCAGCTTCGTCGCCGCGCCGGCG
201  D  G  C  G  G  L  Q  A  G  S  R  L  I  S  F  V  A  A  P  A
661  GCGCCAGTAGACGCTGCCGCGGCGCCGACGACCAGCACGATCACCACGGTCACCGCGCCG
221  A  P  V  D  A  A  A  A  P  T  T  S  T  I  T  T  V  T  A  P
721  GGCCCGCTGCTGCAGCCGCTCAAGGTGGAGGGCGGCGTCGGCTCGTCCGACCAGGAGGAG
241  G  P  L  L  Q  P  L  K  V  E  G  G  V  G  S  S  D  Q  E  E
781  GTGCTGAGCAGCCTCACGCCCGGCAGCTCCGCGGCGCGCGGCGGCGGCGGCGGCGGCGGA
261  V  L  S  S  L  T  P  G  S  S  A  A  R  G  G  G  G  G  G  G
841  GTCGCGGGTCCCTTCGGGCCGGACCAGGGCGATGTCACGTCCTCCCTGCACTGGAGCTAC
281  V  A  G  P  F  G  P  D  Q  G  D  V  T  S  S  L  H  W  S  Y
901  GACGCCGTCGCCGGCATGGAGTTCTTCAAGAACGACGAGGTTGTCTTCGATCTGGACGAC
301  D  A  V  A  G  M  E  F  F  K  N  D  E  V  V  F  D  L  D  D
961  ATTATGGGTTTGAGCTTTTGA
321  I  M  G  L  S  F  *
```

Figure S1. Sequences of nucleotides and deduced amino acids of *OsWRKY45*. The stop codon is marked by an asterisk.

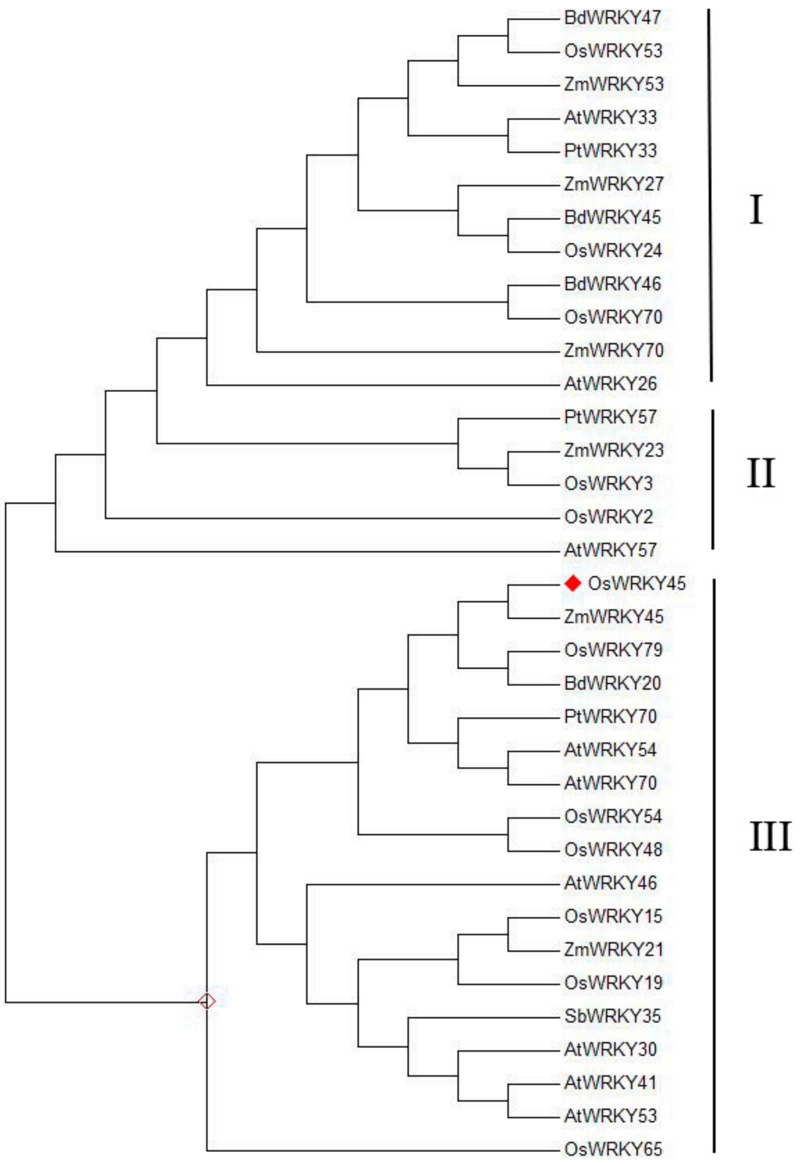

Figure S2. Phylogenetic analysis of WRKY TFs from different species. The unrooted tree was constructed with a neighbor-joining method on the basis of the alignment of protein sequences and confirmation of the tree topology by bootstrap analysis (1000 replicates) were performed with MEGA software (default settings except the replicates of the bootstrap value). Species acronyms are included before the protein name: At, *Arabidopsis thaliana*; Bd: *Brachypodium distachyon*; Pt: *Populus trichocarpa*; Sb: *Sorghum bicolor*; Ta: *Triticum aestivum*; Zm: *Zea mays*. Sequence data in the phylogenic tree can be found in the GenBank/EMBL data libraries under accession number: AtWRKY26 (At5g07100); AtWRKY30 (At5g24110); AtWRKY33 (At2g38470); AtWRKY41 (At4g11070.1); AtWRKY46 (At2g46400); AtWRKY53 (At4g23810); AtWRKY54 (At2g40750); AtWRKY57 (AT1G69310); AtWRKY70 (At3g56400); BdWRKY20 (Bradi1g63220.1); BdWRKY45 (Bradi2g53760.1); BdWRKY46 (Bradi2g22440.1); BdWRKY47 (Bradi2g00280.1); OsWRKY2 (Os10g42850); OsWRKY3 (Os03g55080); OsWRKY15 (Os01g46800.1); OsWRKY19 (Os05g49620.1); OsWRKY24 (Os01g61080); OsWRKY45 (Os05g25770.1); OsWRKY48 (Os05g40060); OsWRKY53 (Os05g27730); OsWRKY54 (Os05g40080); OsWRKY65 (Os12g02470.1); OsWRKY70 (Os05g39720); OsWRKY79 (Os03g21710.1); PtWRKY33 (Potri.013G153400.1); PtWRKY57 (Potri.008G094000.1); PtWRKY70 (Potri.013G090300.1); SbWRKY35 (Sobic.010G045700.1); ZmWRKY21 (GRMZM2G408462_T01); ZmWRKY23 (GRMZM2G018721_T01); ZmWRKY27 (GRMZM2G036703_T01); ZmWRKY45 (GRMZM2G004060_T01); ZmWRKY53 (GRMZM2G012724_T01); ZmWRKY70 (GRMZM2G012724_P01). I, II and III represent group I, group II and group III of WRKY TFs, respectively. The red diamond indicates the gene *OsWRKY45* that is characterized in this paper.

```
OsWRKY45--     1 ATGACGTCATCGATGTCGCCGGCGCCGGCGCCGGCGTACGCGCAGGTGATGGAGGACATG
OsWRKY45-1     1 ATGACGTCATCGATGTCGCCGGCGCCGGCGCCGGCGTACGCGCAGGTGATGGAGGACATG
OsWRKY45-2     1 ATGACGTCATCGATGTCGCCGGCGCCGGCGCCGGCGTACGCGCAGGTGATGGAGGACATG
consensus      1 ************************************************************

OsWRKY45--    61 GAGAAGGGGAAGGAGCTGGCGGCGCAGCTGCAGGGGCTCCTCCGCGACTCGCCGGAGGCC
OsWRKY45-1    61 GAGAAGGGGAAGGAGCTGGCGGCGCAGCTGCAGGGGCTCCTCCGCGACTCGCCGGAGGCC
OsWRKY45-2    61 GAGAAGGGGAAGGAGCTGGCGGCGCAGCTGCAGGGGCTCCTCCGCGACTCGCCGGAGGCC
consensus     61 ************************************************************

OsWRKY45--   121 GGCCGCTTCGTCGACCAGATTCTCCACACCTTCTCCCGGGCGATGCGGGCGCTCGACAAG
OsWRKY45-1   121 GGCCGCTTCGTCGACCAGATTCTCCACACCTTCTCCCGGGCGATGCGGGCGCTCGACAAG
OsWRKY45-2   121 GGCCGCTTCGTCGACCAGATTCTCCACACCTTCTCCCGGGCGATGCGGGCGCTCGACAAG
consensus    121 ************************************************************

OsWRKY45--   181 GCGGCGGTCTCCGCCGCCGGAGGAGAAGGGTCGGAGGTGCAGAGCGAGGTCACCTGCGGG
OsWRKY45-1   181 GCGGCGGTCTCCGCCGCCGGAGGAGAAGGGTCGGAGGTGCAGAGCGAGGTCACCTGCGGG
OsWRKY45-2   181 GCGGCGGTCTCCGCCGCCGGAGGAGAAGGGTCGGAGGTGCAGAGCGAGGTCACCTGCGGG
consensus    181 ************************************************************

OsWRKY45--   241 GGCGGGGCCAGCGCCGGCGGGAAGAGGAAAGCCCCCGCCGCCGACCGGAAGGCCAACTGC
OsWRKY45-1   241 GGCGGGGCCAGCGCCGGCGGGAAGAGGAAAGCCCCCGCCGCCGACCGGAAGGCCAACTGC
OsWRKY45-2   241 GGCGGGGCCAGCGCCGGCGGGAAGAGGAAAGCCCCCGCCGCCAACCGGAAGGCCAACTGC
consensus    241 *********************************************.**************

OsWRKY45--   301 CGCAGGAGGACGCAGCAATCGTCCGGGAATTCGGTGGTCGTCAAGAACCTCGACGACGGC
OsWRKY45-1   301 CGCAGGAGGACGCAGCAATCGTCCGGGAATTCGGTGGTCGTCAAGAACCTCGACGACGGC
OsWRKY45-2   301 CGCAGGAGGACGCAGCAATCGTCCGGGAATACGGTGGTCGTCAAGAACCTCGACGACGGC
consensus    301 ******************************.*****************************

OsWRKY45--   361 CAGGCATGGCGCAAGTACGGGCAGAAGGAGATCCAAAACTCCAAGCACCCAAAGGCCTAC
OsWRKY45-1   361 CAGGCATGGCGCAAGTACGGGCAGAAGGAGATCCAAAACTCCAAGCACCCAAAGGCCTAC
OsWRKY45-2   361 CAGGCATGGCGCAAGTACGGGCAGAAGGAGATCCAAAACTCCAAGCACCCAAAGGCCTAC
consensus    361 ************************************************************

OsWRKY45--   421 TTCCGGTGCACGCACAAGTACGACCAGCTGTGCACGGCGCAGCGGCAGGTGCAGCGCTGC
OsWRKY45-1   421 TTCCGGTGCACGCACAAGTACGACCAGCTGTGCACGGCGCAGCGGCAGGTGCAGCGCTGC
OsWRKY45-2   421 TTCCGGTGCACGCACAAGTACGACCAGATGTGCACGGCGCAGCGGCAGGTGCAGCGCTGC
consensus    421 ***************************.********************************

OsWRKY45--   481 GACGACGACCCGGCGAGCTACAGGGTCACCTACATCGGCGAGCACACCTGCCGGGACCCG
OsWRKY45-1   481 GACGACGACCCGGCGAGCTACAGGGTCACCTACATCGGCGAGCACACCTGCCGGGACCCG
OsWRKY45-2   481 GACGACGACCCGGCGAGCTACAGGGTCACCTACATCGGCGAGCACACCTGCCGGGACCCG
consensus    481 ************************************************************

OsWRKY45--   541 GCCACCGCCCCCATCATCGCGGCGCACGTCATCCACCAGGTCGCCGCCGGCGACAACGAC
OsWRKY45-1   541 GCCACCGCCCCCATCATCGCGGCGCACGTCATCCACCAGGTCGCCGCCGGCGACAACGAC
OsWRKY45-2   541 GCCACCGCCCCCATCATCGCGGCGCACGTCATCCACCAGGTCGCCGCCGGCGACGACGAC
consensus    541 ******************************************************.*****

OsWRKY45--   601 GACGGCTGCGGCGGCCTCCAAGCGGGGTCCCGCCTCATCAGCTTCGTCGCCGCGCCGGCG
OsWRKY45-1   601 GACGGCTGCGGCGGCCTCCAAGCGGGGTCCCGCCTCATCAGCTTCGTCGCCGCGCCGGCG
OsWRKY45-2   601 GACGGCTGCGGCGGCCTCCACGCGGGGTCCCGCCTCATCAGCTTCGTCGCCGCGCCGGCG
consensus    601 ********************.***************************************
```

Figure S3. *Cont.*

```
OsWRKY45--   661 GCGCCAGTAGACGCTGCCGCGGCGCCGACGACCAGCACGATCACCACGGTCACCGCGCCG
OsWRKY45-1   661 GCGCCAGTAGACGCTGCCGCGGCGCCGACGACCAGCACGATCACCACGGTCACCGCGCCG
OsWRKY45-2   661 GCGCCAGTAGACGCTGCCGCGGCGCCGACGACCAGCACGATCACCACGGTCACCGCGCCG
consensus    661 ************************************************************

OsWRKY45--   721 GGCCCGCTGCTGCAGCCGCTCAAGGTGGAGGGCGGCGTCGGCTCGTCCGACCAGGAGGAG
OsWRKY45-1   721 GGCCCGCTGCTGCAGCCGCTCAAGGTGGAGGGCGGCGTCGGCTCGTCCGACCAGGAGGAG
OsWRKY45-2   721 GGCCCGCTGCTGCAGCCGCTCAAGGTGGAGGGCGGCATCGGCTCGTCCGACCAGGAGGAG
consensus    721 ************************************.***********************

OsWRKY45--   781 GTGCTGAGCAGCCTCACGCCCGGCAGCTCCGCGGCGCGCGGCGGCGGCGGCGGCGGCGGA
OsWRKY45-1   781 GTGCTGAGCAGCCTCACGCCCGGCAGCTCCGCGGCGCGCGGCGGCGGCGGCGGCGGCGGA
OsWRKY45-2   781 GTGCTGAGCAGCCTCACGCCCGGCAGCTCCGCGGCGCGCGGCGGCGG------------A
consensus    781 ***********************************************............*

OsWRKY45--   841 GTCGCGGGTCCCTTCGGGCCGGACCAGGGCGATGTCACGTCCTCCCTGCACTGGAGCTAC
OsWRKY45-1   841 GTCGCGGGTCCCTTCGGGCCGGACCAGGGCGATGTCACGTCCTCCCTGCACTGGAGCTAC
OsWRKY45-2   829 GTCGCGGGTCCCTTCGGGCCGGACCAGGGCGATGTCACGTCCTCCCTGCACTGGAGCTAC
consensus    841 ************************************************************

OsWRKY45--   901 GACGCCGTCGCCGGCATGGAGTTCTTCAAGAACGACGAGGTTGTCTTCGATCTGGACGAC
OsWRKY45-1   901 GACGCCGTCGCCGGCATGGAGTTCTTCAAGAACGACGAGGTTGTCTTCGATCTGGACGAC
OsWRKY45-2   889 GACGCCGTCGCCGGCATGGAGTTCTTCAAGAACGACGAGGTTGTCTTCGATCTGGACGAC
consensus    901 ************************************************************

OsWRKY45--   961 ATTATGGGTTTGAGCTTTTGA
OsWRKY45-1   961 ATTATGGGTTTGAGCTTTTGA
OsWRKY45-2   949 ATTATGGGTTTGAGCTTTTGA
consensus    961 *********************
```

Figure S3. cDNA sequence alignment of *OsWRKY45* cloned in this article with *japonica*-derived *WRKY45* (*OsWRKY45*-1) and *indica*-derived*WRKY45* (*OsWRKY45*-2). The asterisk indicates the bases that are identical in the three genes; the dot indicates the bases that are identical in two of the three genes. Non-shaded spaces represent inconsistent bases in the three genes.

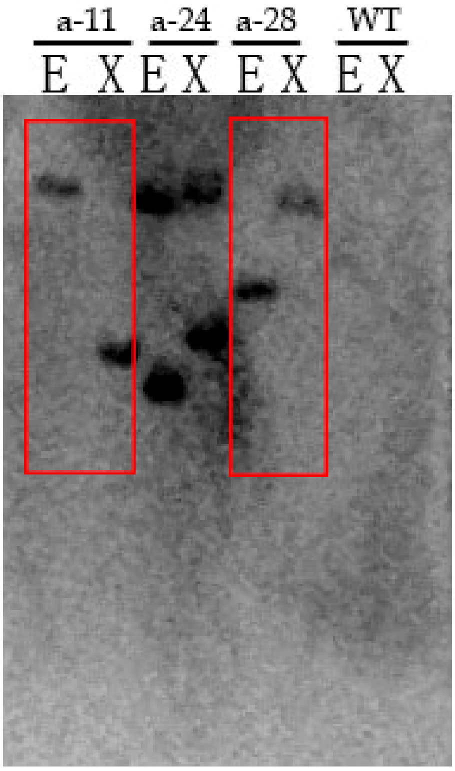

Figure S4. DNA gel-blot analysis of as-*wrky*. Genomic DNA was digested with *Eco*R I (E) or *Xba* I (X). The blot was hybridized with a probe specific for reporter gene *gus*. Two transgenic lines (as-11 and as-28) have a single insertion of the transgene.

```
OsWRKY45        ATG---------------------------ACGTCATCGATGTCGCCGGCGC--------
OsWRKY15        --------------------------------------ATGGCCG---------------
OsWRKY48        ATGGCGCTGATTGCCACTGGTGCTACCGCTACTGCTACTGCGGCGCCGGTGGCTAGCCCG
OsWRKY54        ATGGCGCTCACTATGGTTAGTCTCGCCGC-----------CATCGCCGG---GAAGCC--
                                                         . **

OsWRKY45        -------CGGCGCCGGCGTACGCGCAGGTGATGGAGG---------ACATGGAGAAGGGG
OsWRKY15        ----------CCGGAGCGGACGAGCGGTGCCGCGCGCTGGTGTCCGGCCTGCTGTCGTCG
OsWRKY48        GCTGCTTCTTCCATGGCGTCCG---AGCTGATGGCGCAGGGG------CGGGAGTCCGCC
OsWRKY54        --------CACCGCGGCGTCGGCTCAGCTCGTCGCCGAGGGG------AGGGAGTCGGCG
                          *   .*** . *   .*      *.             . * :*:.

OsWRKY45        AAG----GAGCTGGCGGCGCAGCTGCAGGGGCTCCTCCGCGACTCGCCGGAGGCC-----
OsWRKY15        ATCGACCGGTCTATAAGCATCGCGAG----------------------GTCGT---GCTG
OsWRKY48        ----GCCGTCCTGGAAGCCCTGCTCCATG-GCGCGTC-GC-TCCCGCCGGCGCACGGCGG
OsWRKY54        ----GCCCGCCTGTATGCCCTGCTGGTCG-GCTCGTC-----------GGCGCTCCACGG
                          **. . **   **                         * .*

OsWRKY45        ----GGCCGCTTCGTCGACCAGATTCTCCACACCTTCTCCCGGGCGATGCGGGCGCTCGA
OsWRKY15        CACGGAGGCCGCCGCCGCCGGG----------------------CG---------CCTGA
OsWRKY48        TGCCCACGCCCTCGCCGCCGAGATCCTTCGCTGCTGCGACCGCGCG---------CTCGC
OsWRKY54        CCCTGTGGGCCTCGCCGAGCAGATCCTCCTCTGCTTCGACCGCGCG---------CTCGC
                         *  ** **.  .*                      **         *  *.

OsWRKY45        CAAGGC---GGCGGTCTCCGCCGCCGGAGGA----------------------GAAGGGT
OsWRKY15        C--CCAGCAGGCCGGCGCCG-CGCCTGAGTCGCCGCCGTCCGCCGACGGTAGCGCCGGCT
OsWRKY48        CGCGTTGCGCGCCGGCGGCGATGCCGAA-TC---GTCGTCAGC-----------------
OsWRKY54        CAAGCTGCACGGCGTCAACCTCGCCGGA-GC---TGAGGACGACGACGACGCTGCCGGCA
                *         *  * *  *   *** .*  .

OsWRKY45        C-----------GGAGGTGCAGAGCGAGGTCA--CCTG-CGGGGGCGGG-----------
OsWRKY15        CGGACCTCGGC-------G----CCGACT--------CCCGGTGCCGCGCCAATG-----
OsWRKY48        -------CGACACGAAGCGCAAGCCGGCAACCGCCCAGCCGTCGACGCGCCGGAGGAG--
OsWRKY54        ACGGCAACGGCAGGAAGCGCAAGCCGGGG----------CGTGGTCGTGGCTTGACTGCC
                                  *     **.            **  * ** *

OsWRKY45        --GCCAGCGCCGGCGG----GAAGAGGAAAGCCCCCGCCGCCGACCGGAAGGCCAACTGC
OsWRKY15        ----CGGCGGGGCCGTGCAAGAAGAGGAAGA-------C------------GCT-----G
OsWRKY48        --AAGAGCGACGGCGAGCGGCG-----------------------------GCGGAGCGG
OsWRKY54        GCTTCAGCTGCAGCGAGCTCCAAAAGGATGA-------G------------GGTGAGCAA
                     .**   . **      .                             *

OsWRKY45        CGCAGGAGGACG------CAGCAATCGTCCG---GGAATTCGGTGGTCGTCAAGAACCTC
OsWRKY15        CCCA------AGTGGAGCAAGCAGGTGAAGGTGAGGTCCGTGCAGGACGTCGGCCCCCTC
OsWRKY48        C---GGCGGCGGCGGAGCCGGCGA----------GGGTGGAGAAG-GCGCGG--ACGTCG
OsWRKY54        TGCTGGAGGAAATGGA----GCAA----------GGATTGAGAGG-AAAGCG--ACAATG
                           .        **..          **     *  *  ..  .  ..

OsWRKY45        GACGACGGCCAGGCATGGCGCAAGTACGGGCAGAAGGAGATCCAAAACTCCAAGCACCCA
OsWRKY15        GACGACGGGTTCAGCTGGAGGAAGTACGGGCAGAAGGACATCCTCGGCGCCAAGTACCCA
OsWRKY48        GAGGACGGATTCCTGTGGAGGAAGTACGGGCAGAAGGAGATCAAGAACAGCAAGCACCCG
OsWRKY54        GATGACAAATTTCTGTGGAGGAAGTACGGGCAGAAGGAGATCAAGAACAGCAAGCACCCG
                ** ***..  :    ***.* ***************** ***.: ..*  **** ****.

OsWRKY45        AAGGCCTACTTCCGGTGCACGCACAAGTACGACCAGCTGTGCACGGCGCAGCGGCAGGTG
OsWRKY15        AGAGCCTACTTCCGGTGCACGCACCGGCACACGCAGGGCTGCCACGCCAGCAAGCAGGTG
OsWRKY48        AGGCTCTACTACCGGTGCAGCTACAAGGACGACCATGGCTGCACGGCGACGAAGCAGGTC
OsWRKY54        AGGTTTTACTACCGGTGCAGCTACAAGGACGACCATGGCTGCACGGCGACGAAGCAAGTC
                *..   ****:********   **..* **.. **    ***.. ** .  ..***.**
```

Figure S5. *Cont.*

```
OsWRKY45        CAGCGCT---------------GCGACGACGACCCGGCGAGCTACAGGGTCACCTACATC
OsWRKY15        CAGCG---------CGCGGACGGCG------ACCCGCTGCTCTTCGACGTCGTGTACCAC
OsWRKY48        CAGCAGTCGGAGGAA---------G------ACCCTTCCCTCTACGTCATCACCTACTTC
OsWRKY54        CAGCAGTCGGAGACGGCGGACGACGACACTGCCTCGCCCGTCTACATCATCACCTACTTC
                ****.                   *      .* *      **:*.  .**.  *** :*

OsWRKY45        GGCGAGCACACCTGCCGGGA---CCC---GGCCA---CCGCCCCCATCATCG--------
OsWRKY15        GGCGACCACACCTGCGCGCATGGCGTGCGCTCCGCCGCCGCCGCCATCGACGGCCAGGCC
OsWRKY48        GGCGACCACACCTGCAGCT---GCCAGACGGCCGCCGCCGCCGCCATGGACGACGACGA-
OsWRKY54        GGCGAGCACACCTGCCG------CCATGGGGACGACGCCGCCGCCATGGTCGTCGACGG-
                ***** *********        *       .*.   ***** **** .:**

OsWRKY45        -CGGCGCACGTCATC------CACCAGGTCGCCGCCGGCGA-------CAACGACGACGG
OsWRKY15        GCGGCGAGCGCGGAGCAAAAGCATCA-GCCGACGCCGCCGCAGGAGCAGAACGCCGTCTC
OsWRKY48        -CGACGA----TGA---AAACTCGCA-G------------C------ATTTCGTCATCAA
OsWRKY54        -CGGCGA----GGA---GGAGGATCA-GCTCTCGCCGGCGC------AGATGGTCATCAG
                 **.**.     .:        . ** *            .        :: * *.:*

OsWRKY45        CTGCGG---CGGCCTCCAAGCGGGGTCCCGCCTCATCAGCTTCGTCGCCGCGCCGGCGGC
OsWRKY15        GGTCGCGTTC------------------------------------ACCTCCATG-----
OsWRKY48        CTTCGGGCC-GGCCACCGCG-----AGCCG---CAGC-GGTTCG---CCGCCTCT-----
OsWRKY54        CTTCGCTTCCAGCAACG------------G---CGGC-GATGCTTCTGTGTCCTG-----
                   **

OsWRKY45        GCCAGTAGACGCTGCCGCGGCGCCGACGACCAGCACGA-----TCACCACGGTCACCGCG
OsWRKY15        GCCG-TCGTCAACGCATCGACGTCGTCGCCCTT---------------------------
OsWRKY48        GCTCTACGAC-----GACGGCGACGACGGCGATGTCTGGAGGGAGACGGCGGCCACCCCA
OsWRKY54        GCCGTGC--------TCCGGCGACGACGCCCAGAA-------------------------
                **    .          **.** **:** * :

OsWRKY45        CCGGGCCCGCTGCTGCAGCCGCTCAAGGTGG---AGGGCGGCGTCGGCTCGTCCGACCAG
OsWRKY15        ------------------------CGTGTCC---CCGGCGATGTCGG-----ACTGCCAG
OsWRKY48        CCGTCGTCGAGGCAG-------TCCAGGTGCTCGCCGGAGGGA---G-----ACGGGGAG
OsWRKY54        -CAACAGCGAGACATCGC--ATGAATCGAGCCCGCCGGAGGCGCCGG-----CCGGCGAG
                                        .  *:     . **.*. .   *     .* .  **

OsWRKY45        GAGGAGGTGCTGAGCAGCCTCACGCCCGGCAGCTCCGCGGCGCGCGGCGGCGGCGGCGGC
OsWRKY15        ATTA------------------------------GCTACGAGCTGGGCGGTGGCAGCATG
OsWRKY48        GAATCCGGGGTG----------------------AAGATGAGCAAGGAAG---------A
OsWRKY54        GAAGAACGTCTG----------------------CGGCCGTGCAC--------------A
                .:                                   . * **

OsWRKY45        GGCGGAG----TCGCGGGTCCCTTCGGGCCGGACCAGGGCGATGTCACGTCCTCCCTGCA
OsWRKY15        GCCGGAGTCCGCAACGTGC-CCGACGT----------G---GAGCT--TGCCT-------
OsWRKY48        GCCAGTGG---ACTCGTGT-CCG--GGGCCGTCAG-CG---GTGAGTTCGCCGGCCGACG
OsWRKY54        GCCGCCG------GCGTGT-CTGACGAACCGATCA-TG---GAGTCAACGCCG-CCGGCG
                * *.  *       ** *  *    *           *   .:*      **

OsWRKY45        CTGGAGCTACGACGCCGTCGCCGGCATGGAGTTCTTCAAGAACGACGAGGTTGTCTTCGA
OsWRKY15        --------CCAAGACGAACTCCTCCATGGGAGACGACATGG------AGTTCATGTTCTC
OsWRKY48        TCG---TTTCTTGTTCGTCTCCGGCAATGGAGCCCG--AT------------------CT
OsWRKY54        CCGGAGCTTCTTGC--------GGATCTGAAGCCCA--TG---------GATGGGTGCCT
                         * :            .:  *..  *    :

OsWRKY45        TCTGGACGACATT-----------------------------------------------
OsWRKY15        GCTGGACTCCGATTTCTTGGA------------------------------CACCTA---
OsWRKY48        GCTGGGCTGCTTGAACTGGGATGATGACTTTGGAGACAGCTCGTTCGTCGACGCCGATGA
OsWRKY54        CCTTGACGGCGAGAG------------------------CTTGTTTGGCAT---GGATGA
                 ** *.*  * :
```

Figure S5. *Cont.*

```
OsWRKY45      --------------ATGGGTTTGAGCTTTTGA----------------------------
OsWRKY15      -------------CAAGTACTCCAGCTATTTCTAA-------------------------
OsWRKY48      GTTCATGAATTTCGATGAGATTGATCTGTTTCAAATCTACTCCTAG--------------
OsWRKY54      ACTCGTCTACTTTCATGAGC----------TCTCAGCTGCTCTCGGCCTACTCGATCGAG
                            *:* .            .

OsWRKY45      --------------------
OsWRKY15      --------------------
OsWRKY48      --------------------
OsWRKY54      ACTGGGGTGCGCCAGTATAG
```

Figure S5. cDNA sequence alignment of *OsWRKY45*, *OsWRKY15*, *OsWRKY48* and *OsWRKY15*. Sequence used for *OsWRKY45* antisense transformation is underlined in red. The asterisk under sequences indicates the bases that are identical in the four genes; the colon indicates the bases that have strongly similar properties among sequences; the dot indicates the bases that have weakly similar properties among sequences.

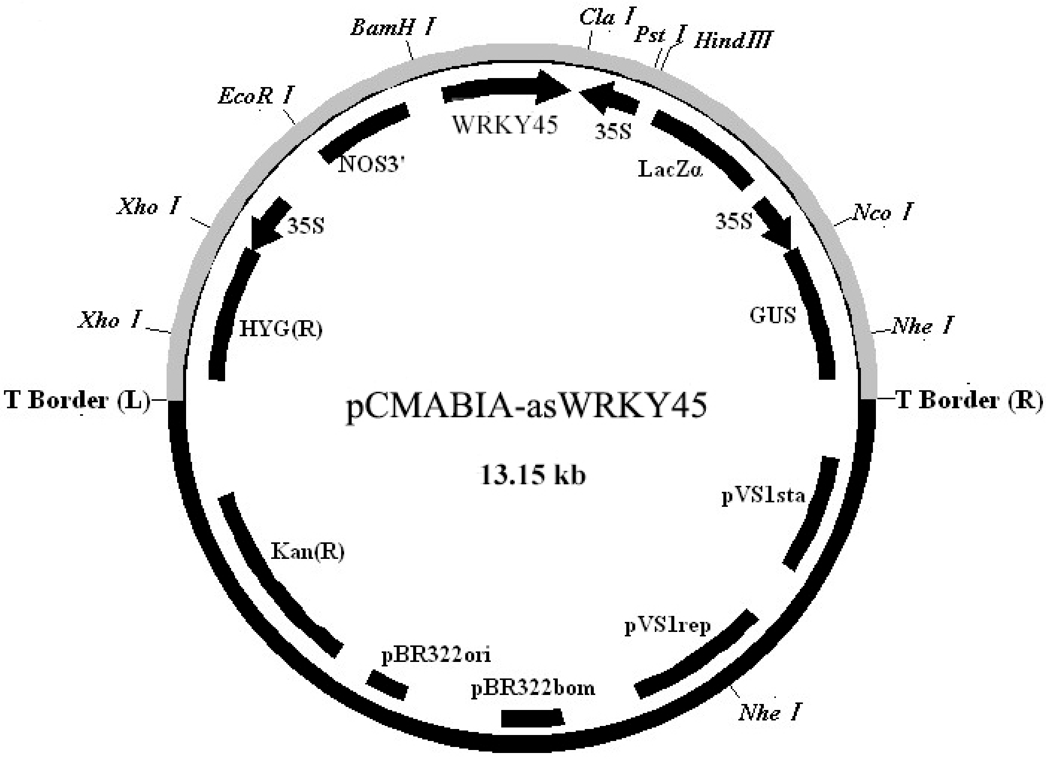

Figure S6. Transformation vectors were used in this study.

Table S1. Primers and probes used for qRT-PCR of target genes.

Gene Name	TIGR ID	Forward Primers(5′–3′)	Reverse Primers (3′–5′)	Probe
ACTIN	Os03g50885	TGGACAGGTTATCACCATTGGT	CCGCAGCTTCCATTCCTATG	CGTTTCCGCTGCCCTGAGGTCC-BHQ1
MPK6	Os06g06090	CGCACGCTCAGGGAGATC	GGTATGATATCCCTTATGGCAACAA	CTCCGCCACATGGACCACGAGAA-BHQ1
MPK3	Os03g17700	CGACTTCGAGCAGAAGGCTCTA	GTTCATCTCGATCGCTTCGTT	ACGAGGACCAAATGAAGCAGCTGAT-BHQ1
WRKY45	Os05g25770	TCGGTGGTCGTCAAGAACC	AAGTAGGCCTTTGGGTGCTT	CAAGTACGGGCAGAAGGAGATCCAA-BHQ1
WRKY53	Os05g27730	AACGGCTGCTCCATGAAGAA	TTGTGTGCGCCCTTGTAGAC	CTCGCCGACGGCCGCATC-BHQ1
WRKY13	Os01g54600	GCGCAAGTACGGCCAGAA	CCTTGGAGCTACTGCACCTGTA	CCCATCAAGGGCTCTCCCTACCCA-BHQ1
OsACS2	Os04g48850	CACCCCGAGGCATCCAT	ATTGGCGATCCTCTTGAACTG	TGCACACCGGAGGGCGTCT-BHQ1
ERF3	Os01g58420	GTTCGCTTTCCTTTCAGAGGATA	GCAGCCTGCTCATAGAAAAAGTAA	CCGTTGCACGTCCAGCAACGC-BHQ3
